# *In vitro* modeling of the female gut microbiome: effects of sex hormones and psychotropic drugs

**DOI:** 10.1128/spectrum.02350-25

**Published:** 2025-10-20

**Authors:** Luana Leao, Galal Ali Esmail, Saba Miri, Walid Mottawea, Riadh Hammami

**Affiliations:** 1 NuGut Research Platform, School of Nutrition Sciences, Faculty of Health Sciences, University of Ottawa 70363, Ottawa, Canada; 2 Department of Biochemistry, Microbiology and Immunology, Faculty of Medicine, University of Ottawa 12365, Ottawa, Canada; Cleveland Clinic Lerner Research Institute, Cleveland, Ohio, USA

**Keywords:** sex hormones, gut microbiota, colon model, in vitro fermentation, psychotropic medication, short-chain fatty acids, sex-specific effects

## Abstract

Sex hormones play a crucial role in shaping gut microbiome composition and metabolism, with significant implications for mental health. This study investigated the effects of sex hormones and the psychotropic drug aripiprazole on the gut microbiome, using a novel *in vitro* colonic fermentation model adapted from the PolyFermS system. Fecal samples from four male and female donors were used to develop sexually divergent models, with the female model subjected to hormonal treatments mimicking different phases of the menstrual cycle. Microbiome composition and short-chain fatty acid (SCFA) metabolism were analyzed. The results demonstrated that sex hormones significantly influenced microbiota structure and diversity, with the female model exhibiting reduced α-diversity and distinct bacterial associations with SCFAs. Hormonal fluctuations across menstrual phases induced specific shifts in bacterial composition, notably increasing Bacteroidota while decreasing Bacillota and Pseudomonadota. In the female model, aripiprazole treatment led to increased microbial diversity and altered SCFA profiles, although the changes in SCFAs were not statistically significant (*P* > 0.05). Differential abundance analysis revealed sex-specific enrichment of bacterial genera, such as *Eubacterium coprostanoligenes* and *Agathobacter*. These findings underscore the importance of considering sex-specific microbiome profiles and hormonal influences when optimizing psychotropic treatments for mental health disorders.

**IMPORTANCE:**

The gut microbiome plays a crucial role in human health, affecting metabolism, immunity, and brain function. However, the role of sex hormones in shaping the gut microbiome composition and metabolism remains largely unexplored. This study introduces a novel *in vitro* colonic fermentation model to investigate the effects of sex hormone fluctuations and psychotropic drug exposure on the gut microbiome. By simulating a sexually divergent human colon environment and mimicking hormonal variations throughout the menstrual cycle, this model provides a controlled setting for studying microbiome response to external stimuli. Our findings revealed that sex hormones, such as estrogen, progesterone, and testosterone, shape microbial diversity and alter the microbiome composition compared to the control group. Additionally, this study examined the effect of psychotropic drug exposure on the microbiota of a simulated female colon, revealing alterations in the microbial composition and metabolism. These results highlight the importance of considering the role of the gut microbiome in drug response, given the widespread use of psychiatric medications, particularly among women. This novel colonic fermentation model offers a valuable tool for studying sex-specific microbiome dynamics and their broader implications for health.

## INTRODUCTION

The gut microbiome is increasingly recognized as a key player in human health, influencing metabolic functions, immune regulation, and neurochemical signaling through the gut-brain axis (1–3). Emerging evidence suggests that sex hormones not only control reproductive functions but also play a significant role in both brain health and composition of the gut microbiome, contributing to sex-specific differences in health and disease (4, 5). These hormonal fluctuations, particularly in females, are associated with changes in gut microbial diversity across the lifespan, including puberty, menstrual cycle, pregnancy, and menopause (6). This hormonal variability not only affects mood and cognitive function but also influences how women metabolize medications, including psychotropic drugs (7). For instance, postmenopausal decreases in estrogen can reduce the levels and effectiveness of certain antipsychotics, such as olanzapine and clozapine, while increasing the levels of others, potentially altering the therapeutic outcomes (8).

Beyond hormonal influences, psychotropic drugs, widely prescribed to manage various psychiatric conditions, are increasingly recognized for their impact on the gut microbiome (9, 10). Studies indicate a higher prevalence of use for these medications among women (15.8%) compared to men (7.3%), a trend particularly evident with antidepressants (11). Psychotropic medications, including antipsychotics and antidepressants, have been shown to alter microbial diversity and metabolic pathways, potentially influencing drug efficacy and side effect profiles (12, 13). Aripiprazole, for example, an atypical antipsychotic prescribed for schizophrenia, bipolar disorder, and major depressive disorder as adjunctive therapy (14), was found to significantly reduce the abundance of Bacillota and Actinomycetota while increasing Pseudomonadota (9). Aripiprazole pharmacokinetics differ between sexes, with women exhibiting higher concentration-dose ratios than men (15). Given that women are more likely than men to use these medications, understanding the interplay between sex hormones, psychotropic drugs, and gut microbiota is crucial for optimizing mental health treatments (11, 13).

Despite increasing interest in hormone-microbiome-drug interactions, mechanistic insights remain limited due to the complexity of *in vivo* systems (16). Alternatively, *in vitro* colonic models present a unique platform that allows for the cultivation of complex microbial communities within controlled settings that closely mimic the human gastrointestinal tract (17). By adapting these models to specific populations, such as males and females, we could explore differences in gut microbiota composition and function between sexes in response to various stimuli (16). Here, we present a sexually divergent *in vitro* colonic fermentation model adapted from the PolyFermS system to investigate the impact of sex hormones and psychotropic drugs on the female gut microbiome (18). This study aimed to (i) examine shifts in the gut microbiome structure and metabolism in response to fluctuations in sex hormones (estrogen, progesterone, and testosterone), (ii) assess microbial composition changes under psychotropic drug exposure, and (iii) elucidate potential microbial biomarkers linked to hormonal and pharmacological modulation.

## MATERIALS AND METHODS

### Fecal inoculum and microbiota immobilization

Four donors provided fecal samples: two men (ages 40 and 36) and two women of reproductive age (ages 30 and 25) with no previous history of mental disorders. This study was conducted under the certificate of ethics approval number H-02-18-347.

Microbiota immobilization was performed using fresh fecal samples encapsulated in gellan-xanthan beads under anaerobic conditions, following the protocol established at N*u*Gut Research Platform (18, 19). This method protects bacteria from environmental stressors and preserves their viability and activity (20). Gel beads (30%) were transferred to a stirred glass reactor containing fresh MacFarlane culture medium.

### Fermentation medium

The fermentation medium, designed to simulate adult human colon chyme (21), was prepared using a combination of carbohydrates, proteins, minerals, and other additives. The carbohydrate sources (g/L) included pectin (2), xylan (2), arabinogalactan (2), guar gum (1), inulin (1), and soluble potato starch (5). Protein sources consisted of mucin (4), casein hydrolysate (3), peptone (5), tryptone (5), yeast extract (4.5), and cysteine (0.8). The mineral composition included KH_2_PO_4_ (0.5), NaHCO_3_ (1.5), NaCl (4.5), KCl (4.5), MgSO_4_ anhydrous (0.6), CaCl_2_·2H_2_O (0.1), MnCl_2_·4H_2_O (0.2), and FeSO_4_·7H_2_O (0.005). Other additives included bile salts (0.4), hemin (0.05), and Tween 80 (1). A filter-sterilized vitamin solution (500 µL/L) was added after autoclaving, containing (μg/L): pyridoxine HCl (10), pantothenic acid (10), p-aminobenzoic acid (5), nicotinic acid (5), thiamin (4), riboflavin (5), biotin (2), folic acid (2), menadione (1), vitamin B12 (0.5), and vitamin K1 (0.0025). The medium components were sourced as described in our recent publication (18).

### Experimental setup and *in vitro* fermentation

**Fig 1:**
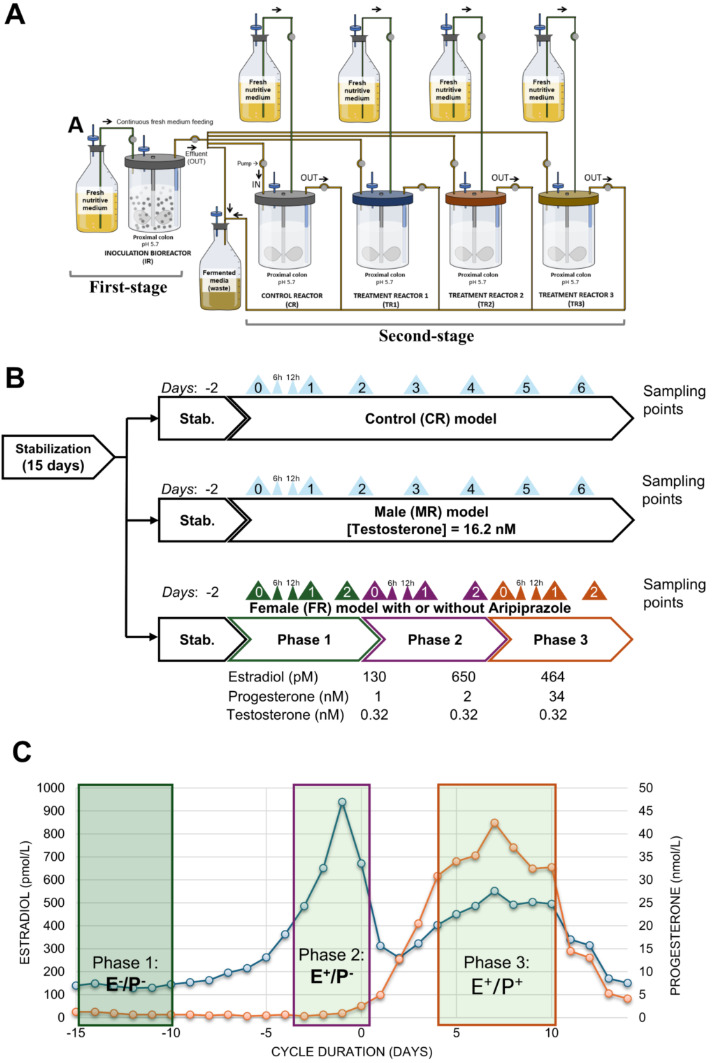
Experimental design of sex-specific colonic fermentation models and study workflow. (**A**) Experimental reactor setup adapted from the *in vitro* PolyFermS fermentation model. The inoculum reactor (IR) contains immobilized donor feces (30% [vol/vol]) to continuously inoculate the system. The setup includes a control reactor (CR) and test reactors (TR1–TR3), each supplied with fresh fermentation medium. Each donor sample, regardless of sex origin, was processed through the same bioreactor setup and operational parameters; the only difference between reactors was the applied treatment. The diagram illustrates the setup for a single donor run as an example. (**B**) Overview of experimental treatments and sampling points. Sampling for the CR and male reactor (TR1) was conducted from days 0 to 6. For the female reactor, without (TR2) or with aripiprazole (TR3), three phases (1, 2, and 3) were defined, each lasting 2 days with sampling points on days 0, 1, and 2. (**C**) Hormone concentrations are outlined for each phase. Estradiol (E) concentrations (blue line) and progesterone (P) concentrations (orange line) are shown over the menstrual cycle, with phases corresponding to the experimental conditions: phase 1 (E–P–), phase 2 (E+*P*–), and phase 3 (E+*P*+).

An *in vitro* simulated colon was used to develop a sexually divergent model to replicate the representative gut microbiota in both men and women. This model consisted of a two-stage design, including an inoculation reactor with immobilized fecal microbiota that was used to inoculate four second-stage reactors that were continuously operated in parallel (Fig. 1A). The impact of testosterone, estrogen, and progesterone on the structure and functionality of the fecal microbiota was evaluated *in vitro* in a colon model mimicking the physiological and microbiological conditions of the human proximal colon (18).

Each fermentation process lasted for 23 days. During the first 48 h, the colonic model operated as a batch culture to allow bead colonization, with the nutritive medium replaced by fresh medium every 12 h. This period was essential for establishing a stable and functional microbial community on the beads because the microorganisms required time to adjust to the new conditions and nutrients provided by the fresh medium. This ensured that the microbial community was established and became active before any subsequent experimental manipulations were performed (22).

Following the initial 48 h, the medium flow was switched to continuous mode for the rest of the experiment. After a 15-day stabilization period, the microbial community in the inoculum reactor (IR) was used to inoculate four second-stage DASGIP bioreactors (Eppendorf, Mississauga, ON): one control reactor (CR, non-treatment control) and three treatment reactors (TR1–TR3), designated for male, female, and female plus psychotropic models, respectively (Fig. 1A). These bioreactors were subjected to different parallel treatment periods, as described below. A working volume of 100 mL was maintained in each second-stage bioreactor by inoculating 5% ([vol/vol]; 0.62 mL/h) IR effluent and adding 95% ([vol/vol]; 11.88 mL/h) fresh fermentation medium. The entire second-stage bioreactor was operated for another 2 days to stabilize the microbial community. Each reactor reproduced the physiological and microbiological conditions of the adult proximal colon (pH 5.7, stirring at 120 rpm, 37°C, and a mean retention time of 8 h) (9). Anaerobiosis was ensured through continuous headspace flushing of N_2_ and CO_2_ at a 0.9:0.1 ratio, and a constant pH of 5.7 was maintained by adding NaOH (2.5 M) (23). Once stabilization was reached in all reactors, the bioreactors were subjected to treatment every 24 h. The hormone concentrations used in each treatment (TR1–TR3) are detailed in Fig. 1B. Female hormone concentrations were determined based on Stricker et al. (24), who identified three distinct phases of the menstrual cycle (E−P−, E+P−, and E+*P*+) according to estradiol and progesterone serum levels (Fig. 1C; Table S1) (24). Hormonal treatments were added to the medium that fed the bioreactors TR2 and TR3, following the 2-day stabilization period. To simulate the menstrual cycle, hormonal concentrations were adjusted every 2 days.

### Psychotropic treatment

The psychotropic aripiprazole was selected following a previous study from our lab, which demonstrated its antimicrobial effects on intestinal bacterial pure cultures and the gut microbiome *in vitro* (9, 25). Stock solutions of aripiprazole at a concentration of 20 mg/mL were prepared according to the manufacturer’s recommendations and filter sterilized (0.22 µm). A final concentration of 400 µg/mL was added to the medium feeding the bioreactor that mimics the female model treated with aripiprazole as a psychotropic (TR3).

### Sampling and sample preparation for analysis

Samples were collected at 0, 12, 24, and 48 h for all the treatment groups. The control (CR) and male (TR1) groups had nine sampling points across days 0−6 (Fig. 1B). For the female (TR2) and female + aripiprazole (TR3) groups, samples were collected during three hormonal phases: E−P−, E+P−, and E+*P*+. The collected samples were separated (centrifugation at 14,000 × *g* for 5 min at 4°C), the pellet was used for metagenomic DNA extraction, and the supernatant was used for short-chain fatty acid (SCFA) analysis.

### Microbiome analysis

The microbial profiles of fecal slurry and fermentation samples were assessed using tag-encoded 16S rRNA gene MiSeq-based high-throughput sequencing. Briefly, genomic DNA was extracted from the pellet of fecal slurry and fermentation samples using a Fast DNA Spin Kit following the manufacturer’s instructions. Briefly, mechanical lysis was performed in two cycles of 40 s each at a speed of 6.0 m/s in a Bead Mill-24 Homogenizer, with a 5-minute cooling period on ice between the two cycles (26). The amount of extracted DNA was quantified by using a Qubit fluorometer. The V3−V4 regions of the 16S rRNA gene were amplified using dual-barcoded primers, and an amplicon library for sequencing was prepared according to the Illumina standard protocol. The amplicon libraries were pooled in equimolar amounts and paired-end sequenced using the Illumina MiSeq platform with the 600 bp MiSeq Reagent Kit v3, following the standard protocol.

### Metabolite analysis using Gas Chromatography combined with a Flame Ionization Detector

SCFA (butyrate, acetate, and propionate) production in fermentation samples from all sub-reactors was assessed using Gas Chromatography combined with a Flame Ionization Detector, following the methods described in previous studies (9, 27). Briefly, supernatants obtained from the fermentation samples underwent centrifugation (at 14,000 × *g* for 30 min at 4°C) followed by filtration using a 0.22 µm filter for sterilization. For quantification, a mixture of formic acid and 2-ethyl butyric acid served as an internal standard, with each sample receiving a concentration of 0.5 mM. Approximately 100 µL of each sample was injected into a capillary column (Stabilwax-DA), and the analysis was performed for 20 min. The resulting peaks were identified and quantified using standards from MilliporeSigma (Oakville, ON, Canada). The results are expressed as the concentration of SCFAs in millimolar (mM) units. All samples underwent duplicate analyses (two technical measurements).

### Bioinformatics analysis

The V3−V4 region sequences were processed using the Qiime2 pipeline (version 2023.2) (28). DADA2 with q2-dada2 plugin was employed for quality control, truncation, and denoising of reads, resulting in an amplicon sequence variant feature table. Taxonomic annotation was performed using the q2-feature-classifier plugin with a pre-trained classifier for regions V3−V4 based on Silva database (release 13.8) (29). The plugin q2-phylogeny was used to obtain a rooted phylogenetic tree. Downstream analyses were performed using “*R*” (v.4.3.1) (https://www.R-project.org/). Relative abundances at the phylum and family taxonomy levels were visualized using line plots to illustrate alterations over time. The presented data represent shifts from baseline (time 0); the time point was compared to the corresponding baseline within the same phase. Alpha diversity was estimated using the observed features and the Shannon and Simpson metrics after rarefaction to the lowest sequencing depth (18,367) using the function “rarefy_even_depth” within the phyloseq package. Beta diversity was calculated using the Bray-Curtis distance and visualized using principal coordinate analysis (PCoA). The contribution of different treatments to the diversity of the gut microbiota community was assessed by the Bray-Curtis distance matrix, using pairwise permutational multivariate analysis of variance with 999 permutations (30). To identify differential taxa among the different treatments, a linear regression model was used to determine the relative abundance of taxa at various levels using linear models for differential abundance analysis (LinDA) (31). The results were considered significant if the *P*-value was <0.05.

### Statistical analysis

The Kruskal-Wallis test with Dunn post-hoc test (FDR-corrected) was applied when the statistical analysis was required to compare differences between the control, female, male, and psychotropic groups using the rstatix package (v. 0.7.2). Data from gas chromatography analyses were analyzed using GraphPad Prism v8.3 (GraphPad Software, USA) to assess the significance of results among treatments simultaneously and among different time points within each treatment. Data are expressed as mean ± SDs of four biological replicates, and an ANOVA test with Bonferroni post-hoc test for multiple comparisons was applied (*P*-value < 0.05).

## RESULTS

### Sex hormones significantly shaped the microbiota structure and diversity

**Fig 2:**
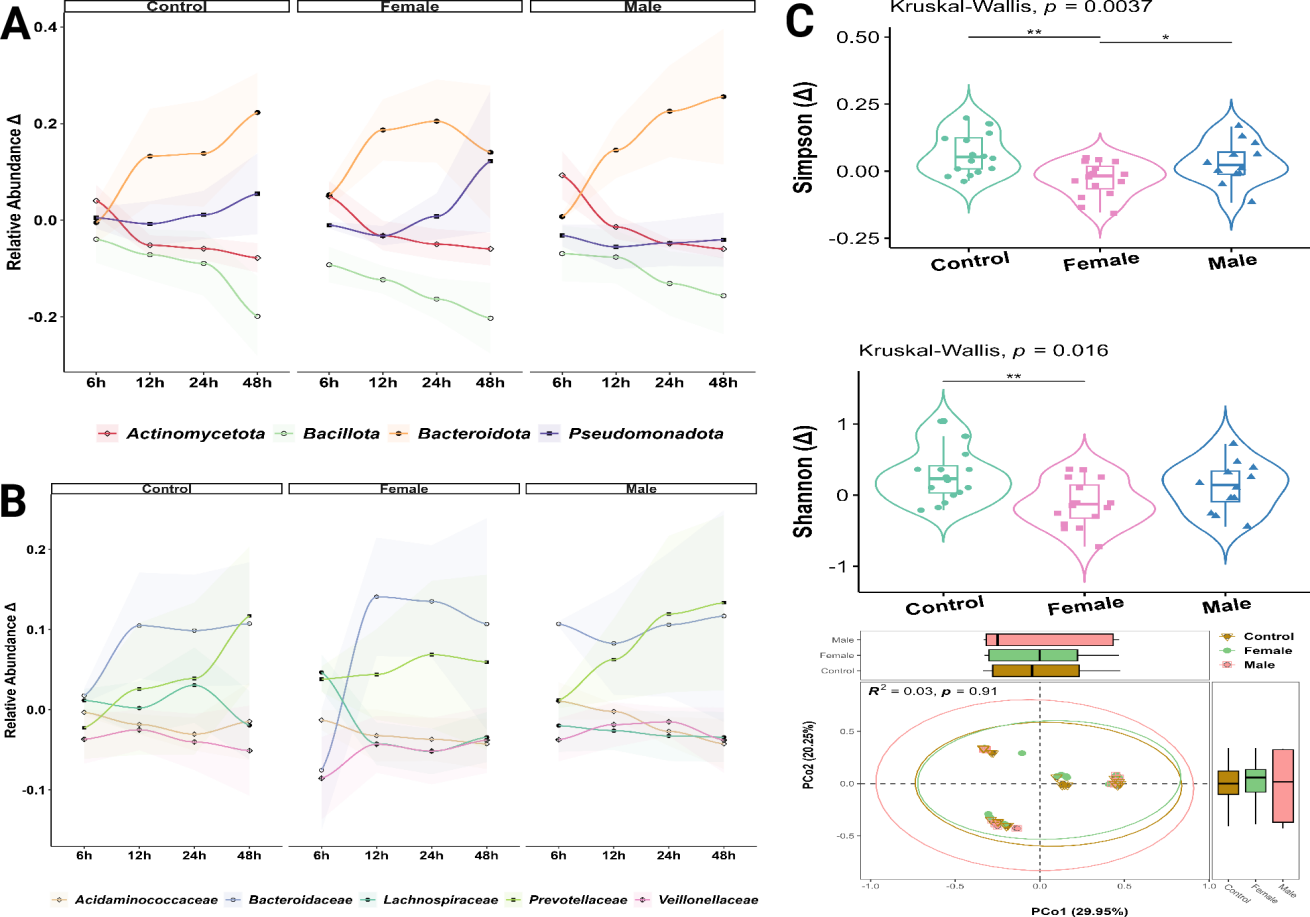
Effects of sex hormones on gut microbiome composition and diversity in colonic fermentation models. (**A**) Line plots illustrate the changes in the relative abundance of the dominant phyla across female, male, and control groups over the first 2 days of fermentation (phase 1). The relative abundance reflects the shift (Δ) from baseline (time 0). (**B**) Line plots display the relative abundance changes in the most prevalent bacterial families over time, showing shifts (Δ) relative to the baseline abundance at time 0. Values in A and B represent the average across all donors, and the shaded areas around the lines indicate the SD (±SD). (**C**) Highlight α-diversity metrics, including changes in Simpson’s Index and Shannon’s Index, with additional analysis of β-diversity using PCoA. Statistical significance is denoted as *P < 0.05 and **P ≤ 0.01.

The shift in microbiota composition over time was compared between the study groups during the first 2 days of fermentation. At the phylum level, the dominant phylum Bacteroidota showed an increase over time in all groups except at time 48 h in the female group (Fig. 2A). In contrast, the relative abundances of Bacillota and Actinomycetota dropped sharply from 12 to 48 h, particularly Bacillota, in all study groups. The relative abundance of Pseudomonadota was unstable at an early fermentation time of 6−12 h, followed by an increase at 24 h and 48 h (Fig. 2A). At the family level, the most abundant families were Acidaminococcaceae, Bacteroidaceae, Lachnospiraceae, Prevotellaceae, and Veillonellaceae. Almost all of the compared groups exhibited an increase in the relative abundance of Bacteroidaceae and Prevotellaceae over time. However, at 48 h, the female group showed a dramatic decrease in the abundance of Bacteroidaceae after stability between 12 and 24 h (Fig. 2B). Veillonellaceae consistently declined in relative abundance over time across all groups (control, female, and male; Fig. 2B).

The α-diversity was measured using three metrics: Observed, Shannon, and Simpson, and the shift from baseline (Time 0) was calculated and statistically investigated. After 2 days of fermentation, we found that the female group had significantly lower α-diversity than the control group, as indicated by the Kruskal–Wallis *P*-value for Shannon and Simpson’s by 0.016 and 0.0037, respectively. Moreover, the female group showed lower observed features (data not shown), Shannon entropy, and Simpson’s diversity compared to the control (*P* < 0.05, observed index; *P* ≤ 0.01, Shannon entropy and Simpson’s index; Fig. 2C). Additionally, the female group had lower Simpson’s index values than the male group (*P* < 0.05; Fig. 2C). Despite significant differences in the α-diversity metrics, the β-diversity analysis revealed no discernible differences between the study groups (Fig. 2C).

SCFA levels did not differ significantly between the groups. However, distinct bacterial associations with SCFAs were observed at specific time points. In both control (Fig. S1A) and female (Fig. S1A) groups, various bacterial families were associated with different SCFAs at specific time points. In the control group, Bacteroidaceae and Veillonaceae were negatively associated with butyrate at 6 h, while Lachnospiraceae and Ruminococcaceae were positively associated (Fig. S1A). By 12 h, Acidaminococcaceae exhibited a positive association with both propionate and butyrate, whereas Bacteroidaceae continued to have a negative association with acetate (Fig. S1A). At 24 h, Bacteroidaceae remained negatively associated with acetate, and Lachnospiraceae displayed positive associations with both propionate and butyrate (Fig. S1A). Acidaminococcaceae was positively associated with acetate and propionate, Enterobacteriaceae was associated with butyrate, and Veillonaceae was negatively associated with both acetate and propionate at 48 h (Fig. S1A). In the female group, Bacteroidaceae were positively associated with acetate at 6 h (Fig. S1B), and Enterobacteriaceae were positively associated with propionate. At 12 h, Bifidobacteriaceae and Veillonellaceae were positively associated with both propionate and butyrate, whereas Oscillospiraceae and Ruminococcaceae were negatively associated with propionate and butyrate (Fig. S1B). Finally, at 48 h, Bifidobacteriaceae and Coriobacteriaceae were positively associated with both propionate and butyrate (Fig. S1B).

**Fig 3:**
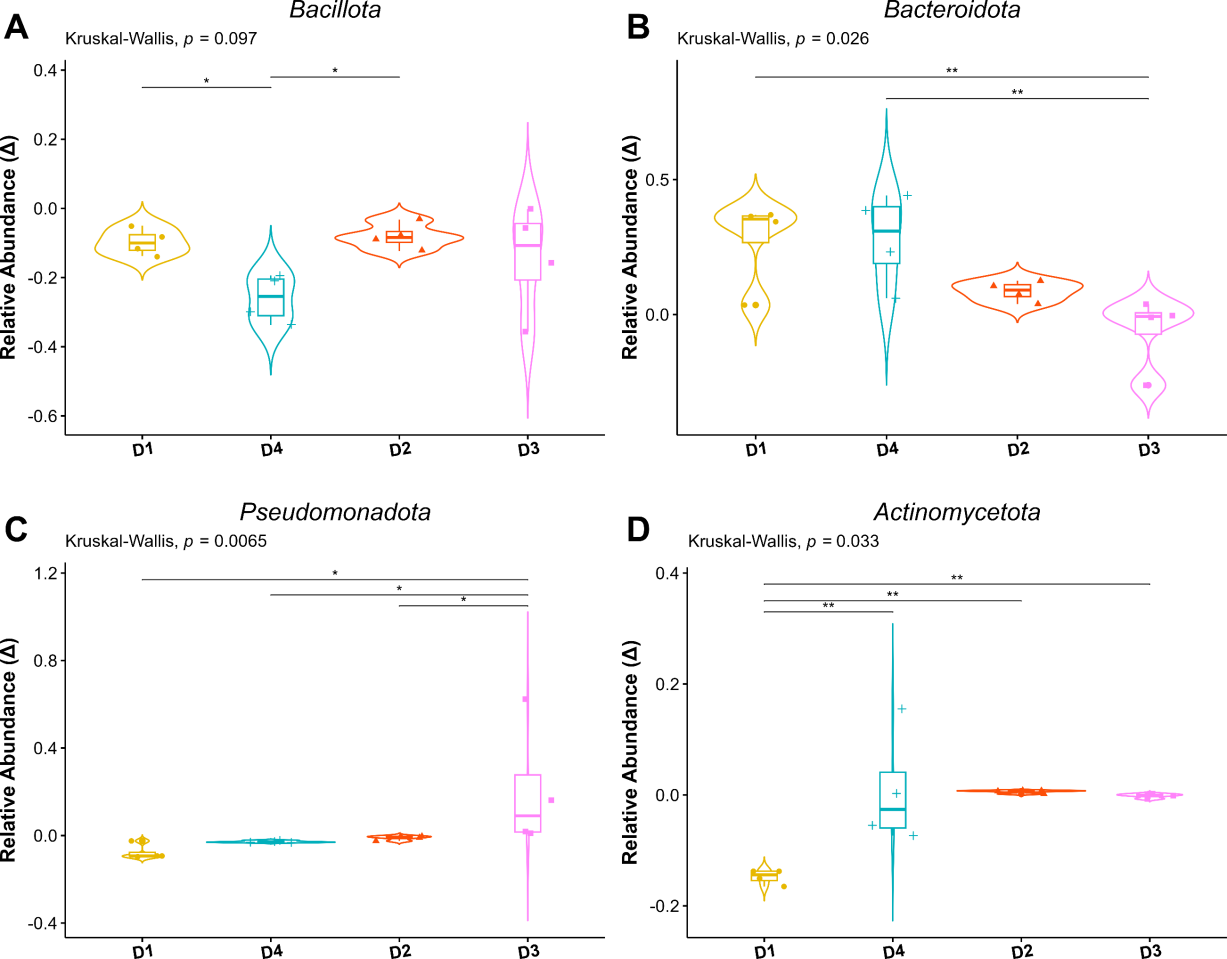
Variations in microbiome composition between male and female donors during the first 48 h of fermentation (phase 1). The relative abundances of the most abundant phyla are shown: Bacillota (**A**), Bacteroidota (**B**), Pseudomonadota (**C**), and Actinomycetota (**D**). Each data point represents an individual time point measurement within this phase. Male donors are represented by D1 and D4, while female donors are represented by D2 and D3. Statistically significant differences are marked as *P < 0.05 and **P ≤ 0.01.

We analyzed the influence of donor sex on microbiota composition in the female model. Kruskal-Wallis analysis revealed significant differences in the abundance of Bacteroidota, Actinomycetota, and Pseudomonadota among donors (*P* < 0.05), while Bacillota abundance remained relatively comparable across all individuals (*P* > 0.05; Fig. 3A through D). However, Bacillota abundance was higher in D1 and D2 than in D4, as indicated by the *P*-value (<0.05; Fig. 3A). Notably, Bacteroidota was significantly higher in male than in female donors (*P* ≤ 0.001; Fig. 3B). Conversely, Pseudomonadota levels were elevated in female donors, particularly on D3 (*P* < 0.05; (Fig. 3C). Actinomycetota abundance was also relatively high in female donors, albeit with a large variation between male donors (Fig. 3D).

### Gut microbiota shifts due to hormonal fluctuations across menstrual phases

**Fig 4:**
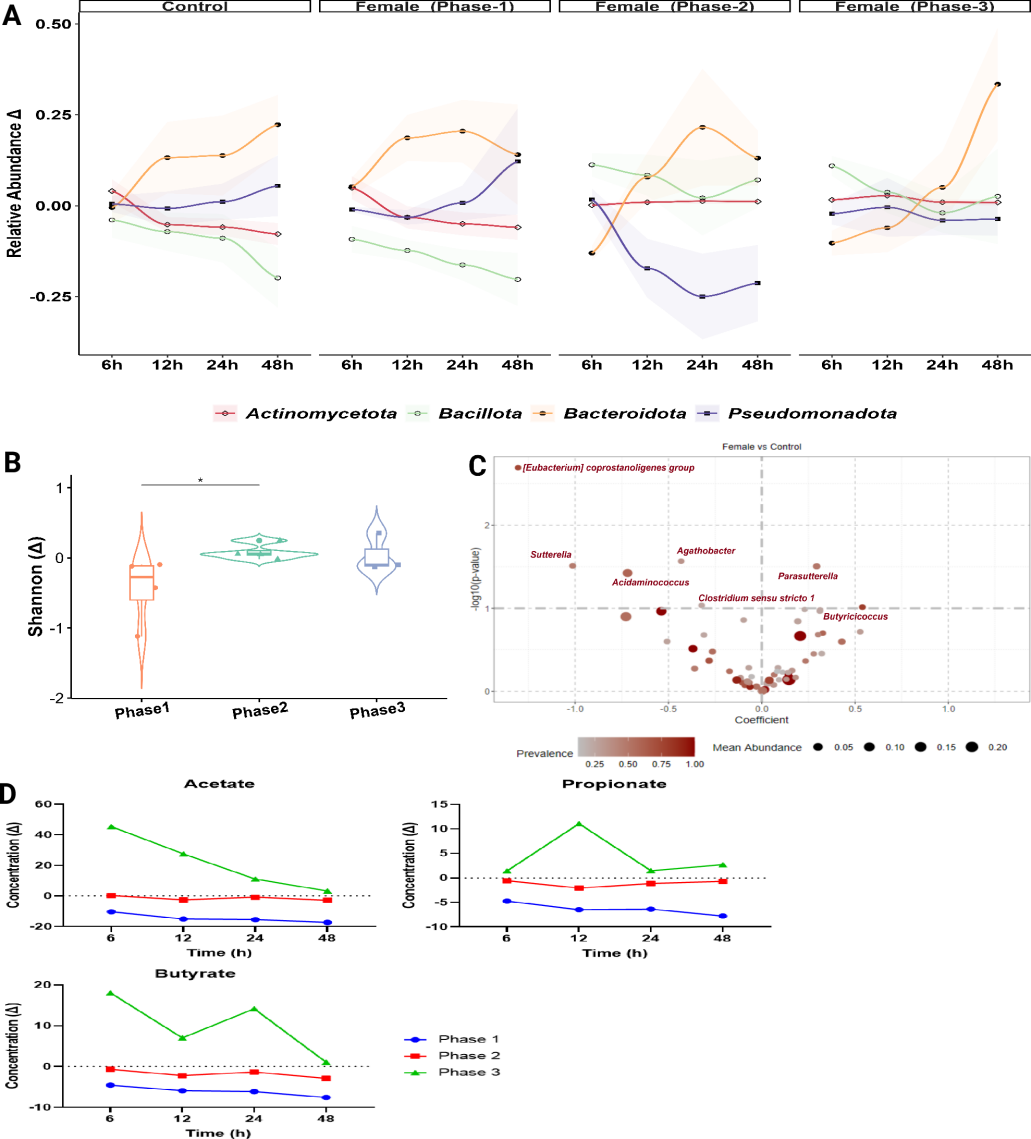
Effects of hormonal fluctuations on gut microbiome diversity and metabolism across menstrual cycle phases in the feminine colonic model. (**A**) Relative abundance of bacterial taxa across control and female model during phases 1−3 of the menstrual cycle, showing changes (Δ) relative to baseline at time 0. Values represent the average across all donors, and the shaded areas around the lines indicate the SD (±SD). (**B**) Shannon Diversity metrics illustrate changes in microbial diversity by cycle phase, expressed as the shift (Δ) from time 0 at 48 h. (**C**) Volcano plot identifies significant genus-level taxa differences between treatment groups and the control at phase 1, determined using the LinDA linear model. (**D**) Line plots display SCFA concentration (mM) changes (Δ SCFA = concentration at time point—baseline) over time. Show SCFA profiles for each cycle phase in the female model. Statistically significant differences are noted as *P < 0.05.

Instability in phylum-level abundance across time points (6, 12, 24, and 48 h) was observed in the control group and across phases in the female model. In both groups, Bacteroidota consistently increased over time, while Bacillota showed a sharp decrease during phase 1 in the female model and remained relatively stable in phases 2 and 3. Actinomycetota maintained a relatively stable abundance, with only minor increases in the female model during phases 2 and 3, whereas Pseudomonadota demonstrated the greatest variability, particularly in the female model, initially decreasing and later recovering, except for phase 2 where it decreased sharply (Fig. 4A). After normalizing the data with a rarefaction approach, alpha diversity was investigated using Observed, Shannon, and Simpson’s metrics. The Observed and Simpson’s metrics were not significant at any time point in any of the three phases. However, a large difference in Shannon diversity (Δ) between phase 1 and phase 2 at 48 h (*P* < 0.05) was found, indicating a notable shift in the microbial community composition and diversity over time (Fig. 4B).

Differential abundance (DA) analysis was performed at the genus level after filtering sequences to remove low-abundance reads (frequency <10) and those present in <10% of samples. The findings revealed significantly different shifts in the gut microbiome composition of females compared to that of the control group (Fig. 4C). DA findings revealed significant enrichment of *Eubacterium coprostanoligenes*, *Agathobacter*, *Sutterella*, and *Acidaminococcus* in the female group compared to that in the control group (*P* = 0.002, *P* = 0.02, and *P* = 0.03, respectively). In contrast, *Parasutterella* was significantly enriched in the control group (*P* = 0.03; Fig. 4C). A comparative analysis of SCFA concentrations across the study groups revealed distinct temporal profiles. In the female group, SCFA concentrations decreased sharply during phase 3 compared to the other phases, showing a more pronounced reduction over time for all three SCFAs (Fig. 4D).

### Impact of psychotropic medication on the gut microbiome in the developed feminine colonic model

**Fig 5:**
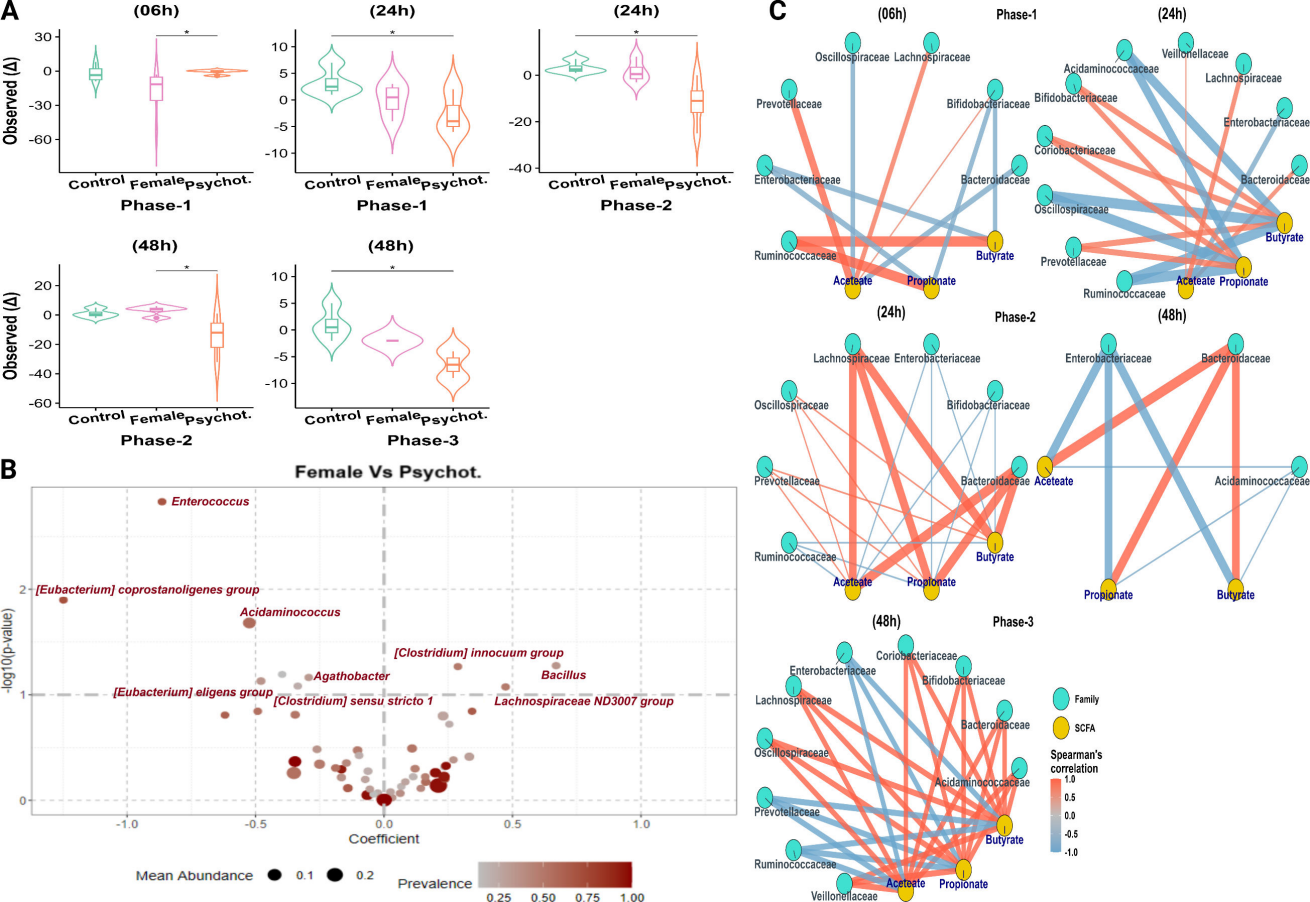
Effects of psychotropic medication on gut microbiome diversity and metabolism across menstrual cycle phases in the feminine colonic model. (**A**) α-diversity between study groups during the menstrual cycle phases. (**B**) DA between female and psychotropic models at phase 1. (**C**) The circular networks display Spearman’s correlations between the most abundant bacterial families (turquoise nodes) and SCFAs (golden nodes) across the psychotropic group during the three phases. Positive correlations are shown in red, negative correlations in blue, with line thickness representing correlation strength. Changes (Δ) in abundance and SCFA concentrations are relative to baseline (time 0). Asterisks indicate statistical significance (*P < 0.05).

The analysis of α-diversity across study groups during different menstrual cycle phases (6, 12, 24, and 48 h) revealed temporal variations in microbial community composition (Fig. 5A). In phase 1, at 6 h, the psychotropic group exhibited a higher observed index than the female group, while at 24 h, the control group demonstrated higher α-diversity than the psychotropic group. In phase 2, at 24 h, the control group showed higher α-diversity than the psychotropic group, and at 48 h, the female group had a higher observed index than the psychotropic group. In phase 3, at 48 h, the control group exhibited higher α-diversity than the psychotropic group. These findings highlight the impact of psychotropic medication on gut microbiome composition, with significant temporal and group-specific variations observed across menstrual cycle phases.

Differential abundance analysis revealed variations between groups across the three phases (Fig. 5B). When comparing the female group to the psychotropic, *Enterococcus* showed a negative shift (*P* = 0.001), indicating a decrease in its abundance under psychotropic influence in phase 1. Similarly, *Eubacterium coprostanoligenes* group and *Acidaminococcus* exhibited reductions (*P* = 0.01, 0.02, respectively), suggesting a negative impact of psychotropic drugs on these bacteria (Fig. 5B). In phase 1 (06 h), positive correlations included Prevotellaceae with acetate and Ruminococcaceae with propionate and butyrate, whereas negative correlations were minimal, suggesting a predominantly cooperative interaction among bacterial families and SCFAs, with Enterobacteriaceae showing weak negative correlations with propionate and butyrate (Fig. 5C). At 24 h in phase 1, positive correlations increased, particularly for Bifidobacteriaceae, Prevotellaceae, and Coriobacteriaceae, with butyrate and propionate, while negative correlations with propionate and butyrate emerged from Ruminococcaceae, Oscillospiraceae, and Acidaminococcaceae. The network became more complex, with propionate showing stronger connections, especially with Bacteroidaceae. In phase 2 (24 h), strong positive correlations were observed between Bacteroidaceae and butyrate and between Lachnospiraceae and acetate, propionate, and butyrate. At 48 h, Bacteroidaceae showed positive correlations with all three SCFAs, whereas Enterobacteriaceae showed negative correlations. Phase 3 (48 h) reveals an increase in network density, reflecting widespread microbial activity. Multiple bacterial families, including Lachnospiraceae, Bacteroidaceae, Acidaminococcaceae, and Bifidobacteriaceae, showed strong positive correlations with all SCFAs, particularly with acetate and butyrate. Negative correlations became more frequent, indicating potential competitive dynamics, as Ruminococcaceae, Enterobacteriaceae, and Prevotellaceae exhibited inverse relationships with all three SCFAs.

## DISCUSSION

In this study, we introduce a novel *in vitro* model designed to investigate the effects of sex steroid hormones on gut microbiota composition and metabolic activity, as well as microbiome responses to external stimuli, such as psychotropic drugs. This model was developed utilizing a modified PolyFermS fermentation system, which facilitated the maintenance of a stable colonic microbiota during hormonal phases. The experimental setup was motivated by the growing recognition of the role of the gut microbiome in neuroendocrine regulation and the rising use of psychotropic drugs among female populations. By simulating a sexually divergent human colon environment, the study aimed to provide a controlled platform to assess microbiome shifts under physiologically relevant hormonal conditions. Our findings provide evidence that hormonal fluctuations influence gut microbiome diversity and metabolism, supporting the hypothesis that sex hormones serve as key modulators of the gut microbiome.

Our results demonstrated that the gut microbiome of male and female models exhibited differences in bacterial abundance. At the phylum level, Bacteroidota increased in all study groups, except for a decline at 48 h in the female group. Pairwise comparisons revealed differences at 24 h between male and female groups, where Bacteroidota was notably higher in females, while Bacillota increased in males. The observed differences in bacterial abundance between male and female colonic models, particularly the higher levels of Bacteroidota in females and Bacillota in males at 24 h, align with findings from the literature, such as d'Afflitto et al. (32). Their meta-analysis revealed that in healthy women, higher estrogen levels are associated with a greater abundance of Bacteroidota and a reduction in Bacillota, particularly within the Ruminococcaceae family (32). These results contrast with those of several previous studies that reported a lower abundance of Bacteroidota and a higher proportion of Bacillota in females than in males. For instance, Koliada et al. (33) found that the relative abundance of Bacillota and Actinomycetota was significantly higher in females, whereas that of Bacteroidota was significantly lower (33). Similarly, Kaliannan et al. (34) reported that the Bacillota/Bacteroidota ratio was elevated in female mice (34). Further complicating the picture, a study in Spain found that Bacteroidota abundance was lower in men than in women with body mass index (BMI) >33 (35). This variability suggests that factors such as BMI, diet, and overall health could also influence the relationship between sex and microbial composition (36). The discrepancies between our findings and those of previous studies may be attributed to variations in hormonal treatment or the conditions under which the studies were conducted. For instance, the hormonal environment in our study might have been different from that reported in studies showing lower Bacteroidota and higher Bacillota in females. Microbial diversity, as measured by α-diversity, further supports the influence of sex hormones on gut microbiota. The lower Simpson’s index values in the female group suggest that females exhibit less evenness in their microbiota, indicating that certain bacterial taxa dominate the ecosystem more than in males. Despite the differences observed in α-diversity, no significant differences in β-diversity were found between the study groups, indicating that, while the evenness of the microbial communities may vary, the overall composition of microbial species remains similar across the sexes. Notably, these results were obtained in the first 2 days of treatment when bioreactors mimicked the follicular phase with low estrogen and progesterone (E−P−-) levels, potentially influencing the observed microbial patterns. These findings contradict some existing literature on the effects of sex hormones on the gut microbiota. Shin et al. (37) investigated the association between serum sex steroid hormone levels and gut microbiome diversity. They found that, although no significant changes in microbiota richness were observed among groups with varying levels of testosterone or estradiol, significant differences in diversity and evenness were noted. Specifically, men with high testosterone levels and women with high estradiol levels show significantly higher diversity and evenness scores than their medium-level counterparts (37).

In our study, low hormone levels may have contributed to the observed reduction in microbial evenness in females, which is consistent with previous studies showing an inverse relationship between estradiol and α-diversity (38). Additionally, the lack of association between estrogen and diversity in premenopausal women, as observed by Flores et al. (39), may help explain the variability in findings across studies (39). Hormonal context, phase of the menstrual cycle, and individual hormonal levels are likely key factors in determining the impact of estrogen on gut microbiota diversity (40). In the female model, distinct patterns of bacterial associations with SCFAs emerged at different time points. At 6 h, Bacteroidaceae were positively associated with acetate, whereas Enterobacteriaceae were linked to propionate. By 12 h, Bifidobacteriaceae and Veillonaceae were positively correlated with propionate and butyrate, whereas Oscillospiraceae and Ruminococcaceae were negatively associated with these SCFAs. By 48 h, Bifidobacteriaceae and Coriobacteriaceae were positively associated with propionate and butyrate. Despite these associations, SCFA concentrations in the female model were lower than those in all other treatment groups, with a notable decline in every cycle phase, particularly in phase 3 (E+*P*+), which exhibited the most pronounced decrease. This decline in SCFA levels may reflect hormonal effects on microbial metabolic activity and highlight potential disruptions in gut health due to hormonal fluctuations (41). To examine the influence of donor sex on the microbial response to hormones, microbiota from both male and female donors were exposed to female sex hormones, mainly estradiol, in phase 1. Hormonal treatment altered bacterial communities in all donor microbiotas, indicating that the effect occurred regardless of the donor’s original sex. Although the original sex of the donor still affected the microbial response, hormonal treatment induced consistent directional changes, such as a decreasing trend in Actinomycetota across both male and female donors. However, the extent and timing of these changes can vary, highlighting the need for further research to understand and optimize the influence of sex hormones on microbial profiles.

Differential abundance analysis at the genus level further highlights the sex-specific influence of hormones. For example, the female bioreactor exhibited enrichment of *Eubacterium coprostanoligenes*, *Agathobacter*, *Sutterella*, and *Acidaminococcus*, whereas *Parasutterella* was significantly enriched in the control group in phase 1. These findings are consistent with those of previous studies that have highlighted the influence of estradiol on microbial composition. Ortiz-Alvarez De La Campa et al. (42) observed that increased estradiol levels were significantly associated with *Eubacterium coprostanoligenes* in mice, supporting our observation of estradiol enrichment in the female model (42). Similarly, Zhou et al. (43) reported a positive correlation between *Sutterella*, *Agathobacter*, and estradiol levels in obese women with polycystic ovary syndrome (PCOS) (43). However, Wu et al. (44) found a positive correlation between *Parasutterella* and estradiol, which is in contrast to our results that showed a higher abundance in the control group (44). In every phase of the cycle, female hormones caused sharper increases in Bacteroidota and more dramatic declines in Pseudomonadota and Bacillota compared to the control, which maintained a more gradual and steady bacterial profile. Notably, the female model demonstrated higher peaks in Bacteroidota and a decline in Bacillota during phases 2 (E+P−) and 3 (E+*P*+), when estrogen levels were elevated. These findings suggest that hormonal fluctuations play a critical role in driving specific shifts in the bacterial composition. This aligns with Shin et al. (37), who reported that higher estradiol levels in healthy women correlated with increased Bacteroidota abundance and reduced Bacillota abundance (37), reinforcing the idea that sex hormones significantly influence the gut microbiome dynamics, particularly throughout the menstrual cycle. Interestingly, despite significant differences in α-diversity, β-diversity analysis revealed no discernible differences between groups or cycle phases. This suggests that, although the overall diversity of bacterial species varied, the community composition remained relatively similar across the different study groups. The SCFA concentrations in the female model were lower than those in all other treatment groups, with a notable decline in every cycle phase, particularly in phase 3 (E+*P*+), which exhibited the most pronounced decrease. This decline in SCFA levels may reflect hormonal effects on microbial metabolic activity and highlight potential disruptions in gut health due to hormonal fluctuations (41).

We observed that psychotropic medication significantly affected gut microbiota, leading to increased microbial diversity and evenness within the bioreactor. This finding aligns with a recent meta-analysis by Minichino et al. (45), who reported that treatment with antipsychotics and antidepressants is associated with significant changes in gut microbiome diversity, taxonomy, and functionality (45). They also found that baseline gut microbiome parameters were linked to subsequent therapeutic responses, highlighting the importance of initial microbial profiles in predicting treatment outcomes (45). The instability in the relative abundance of major phyla, particularly the increase in Bacteroidota and decrease in Bacillota and Actinomycetota, suggests that psychotropic medication may selectively inhibit the growth of certain bacterial populations. This observation is consistent with the findings of Pan et al. (46), who studied longitudinal changes in the microbiome of children during treatment with atypical antipsychotics, including aripiprazole. Their research also indicated changes in the composition of the microbiota over time (46). Furthermore, Flowers et al. (47) reported significant differences in the gut microbiota communities between patients treated with atypical antipsychotics (including aripiprazole) and those who were not (47). In their study, treated women exhibited decreased species diversity compared with the non-treated group, whereas men showed no significant differences between treatment groups (47). This sex-specific response to antipsychotic treatment reinforces our findings of higher α-diversity in the psychotropic group, particularly during the early fermentation phases, despite the suppression of taxa such as *Enterococcus*.

A notable observation from our study is the impact of psychotropic medication on SCFA production, which is a crucial aspect of the gut microbiome function. In our study, acetate levels increased initially but declined after 24 h, while propionate and butyrate levels steadily increased throughout the fermentation period. This shift in SCFA profiles may have important implications for gut health. SCFAs such as acetate, propionate, and butyrate are vital metabolites produced by gut bacteria through fermentation of dietary fibers and resistant starches. These SCFAs contribute to energy metabolism, regulate immune responses, and maintain the integrity of the gut barrier (48). Our results are consistent with the findings of Hua et al. (49), who highlighted that ketamine and its metabolites can enhance antidepressant effects by promoting microbiota associated with SCFAs (49). Lu et al. (50) demonstrated that antidepressant medications exert therapeutic effects by interacting with the gastrointestinal microbiome and its metabolites (50). Furthermore, Singh et al. (51) reviewed evidence showing that antipsychotic drugs can disturb the intestinal barrier, intensify gut-associated lymphoid tissue activity, and reduce SCFA synthesis (51), which aligns with our observation of SCFA profile changes.

Aripiprazole, a partial dopamine agonist, is widely prescribed for conditions such as schizophrenia, bipolar disorder, and, in some cases, major depressive disorder (MDD) (52). These conditions, particularly in women, often intersect with hormonal fluctuations that can modulate both psychiatric symptoms and treatment responses (53, 54). Estrogen and progesterone are known to influence not only the dopaminergic pathways (5, 55) but also, as observed in this and past studies, the gut microbiota, which together play critical roles in mental health (56, 57). This interaction raises important considerations for women with psychotic disorders, who are a significant clinical population taking aripiprazole. For these individuals, the effects of aripiprazole on the gut microbiota may be compounded by sex hormone-driven changes in the gut-brain axis. These results suggest that psychotropic medications may need to be tailored based on the sex-specific microbiome and hormonal status of patients (58). The observed differences between male and female gut microbiomes, often referred to as the “microsexome” (59), indicate that treatments that are effective for one sex may have unintended or less effective outcomes for the other. The “microsexome” not only influences gut composition but also plays a crucial role in regulating immune processes and driving sex differences in disease manifestation (60). For instance, the reduced abundance of beneficial SCFA-producing bacteria in females undergoing psychotropic treatment raises concerns about the long-term impact of these medications on gut health, potentially leading to dysbiosis or metabolic complications (49, 51). As a result, treatments such as psychotropic medications or those aimed at manipulating the microbiome, including fecal microbial transplants, may need to be carefully adjusted based on the patient’s sex to optimize the therapeutic outcomes.

Although fermentation bioreactor models are valuable tools for studying the gut microbiome, they have several limitations. A significant challenge is the inability to fully replicate the complexities of the human gut environment. For instance, these models do not capture key physiological factors such as the movement of the gut (peristalsis), protective mucus layers, or direct interactions between gut microbes and host tissues (61). These limitations indicate that although the model offers controlled conditions for studying microbial dynamics, it may not account for all aspects of gut function and its interaction with microbiota. Despite these limitations, the *in vitro* fermentation model offers several advantages, making it an effective tool for studying the gut microbiome. This system allows for controlled manipulation of different variables, enabling researchers to simulate the specific physiological conditions of both male and female guts (62). The use of immobilized fecal microbiota enhances stability and microbial viability, closely mimicking the environment of the colon (20). Additionally, the continuous mode of operation and parallel reactor design ensure reproducibility and enable long-term studies, providing valuable insights into microbial community dynamics and functional outputs such as SCFA production (62).

This study highlights the need to consider sex differences in the microbiome when prescribing psychotropic medications. Future research should focus on understanding the molecular mechanisms through which sex hormones and psychotropic drugs interact with the microbiome. Additionally, hormonal fluctuations, especially those related to the menstrual cycle or menopause, may further influence microbial responses, thereby emphasizing the importance of hormone-informed treatment strategies. By considering microbiome composition, hormonal status, and sex-specific responses, future psychiatric treatments could achieve more precise and effective results, reduce the risk of gut dysbiosis, and improve overall mental health outcomes.

## Data Availability

All data generated or analysed during this study are available from the corresponding author on reasonable request. The 16S rRNA Illumina amplicon sequencing data are publicly available in the NCBI SRA database under the accession number PRJNA1267560.

## SUPPLEMENTAL MATERIAL

Table S1 and Figure S1 spectrum.02350-25-s0001.pdf Table S1: Hormone concentrations for each hormonal phase. Fig. S1: Correlation heatmaps depict Spearman’s correlation coefficients between the most abundant bacterial families and SCFAs in control (A) and female (B) simulators at four time points (6 h, 12 h, 24 h, and 48 h).

ASM does not own the copyrights to Supplemental Material that may be linked to, or accessed through, an article. The authors have granted ASM a non-exclusive, world-wide license to publish the Supplemental Material files. Please contact the corresponding author directly for reuse.
