# Supplementary material for: In vitro modeling of the female gut microbiome: effects of sex hormones and psychotropic drugs
Source: Microbiol Spectr. 2025 Oct 20;13(12):e02350-25. doi: 10.1128/spectrum.02350-25 (PMC12671099; doi:10.1128/spectrum.02350-25)
Supplement: Table S1 and Figure S1 — Table S1: Hormone concentrations for each hormonal phase. Fig. S1: Correlation heatmaps depict Spearman’s correlation coefficients between the most abundant bacterial families and SCFAs in control (A) and female (B) simulators at four time points (6 h, 12 h, 24 h, and 48 h). [file spectrum.02350-25-s0001.pdf]

Supplementary Materials for

In vitro modeling of the female gut microbiome: Effects of sex hormones and psychotropic drugs

The file includes:

Supplementary Table S1

Supplementary Figures S1

Table S1. Hormone concentrations for each hormonal phase

Female gut	E-P- (Phase 1)	E+P- (Phase 2)	E+P+ (Phase 3)	Reference
**Estradiol (pmol/L)**	130	650	464	Stricker et al. (2006)
**Progesterone (nmol/L)**	1	2	34	
**Testosterone (nmol/L)**	0.32	0.32	0.32	Skiba et al. (2019)
**Male gut**				
**Testosterone (nmol/L)**	16.2	16.2	16.2	Zhu et al. (2022)

References

1. Stricker R, Eberhart R, Chevailler M-C, Quinn FA, Bischof P, Stricker R. 2006. Establishment of detailed reference values for luteinizing hormone, follicle stimulating hormone, estradiol, and progesterone during different phases of the menstrual cycle on the Abbott ARCHITECT® analyzer. Clinical Chemistry and Laboratory Medicine (CCLM) 44.

2. Skiba MA, Bell RJ, Islam RM, Handelsman DJ, Desai R, Davis SR. 2019. Androgens During the Reproductive Years: What Is Normal for Women? The Journal of Clinical Endocrinology & Metabolism 104:5382–5392.

3. Zhu A, Andino J, Daignault-Newton S, Chopra Z, Sarma A, Dupree JM. 2022. What Is a Normal Testosterone Level for Young Men? Rethinking the 300 ng/dL Cutoff for Testosterone Deficiency in Men 20-44 Years Old. Journal of Urology 208:1295–1302.

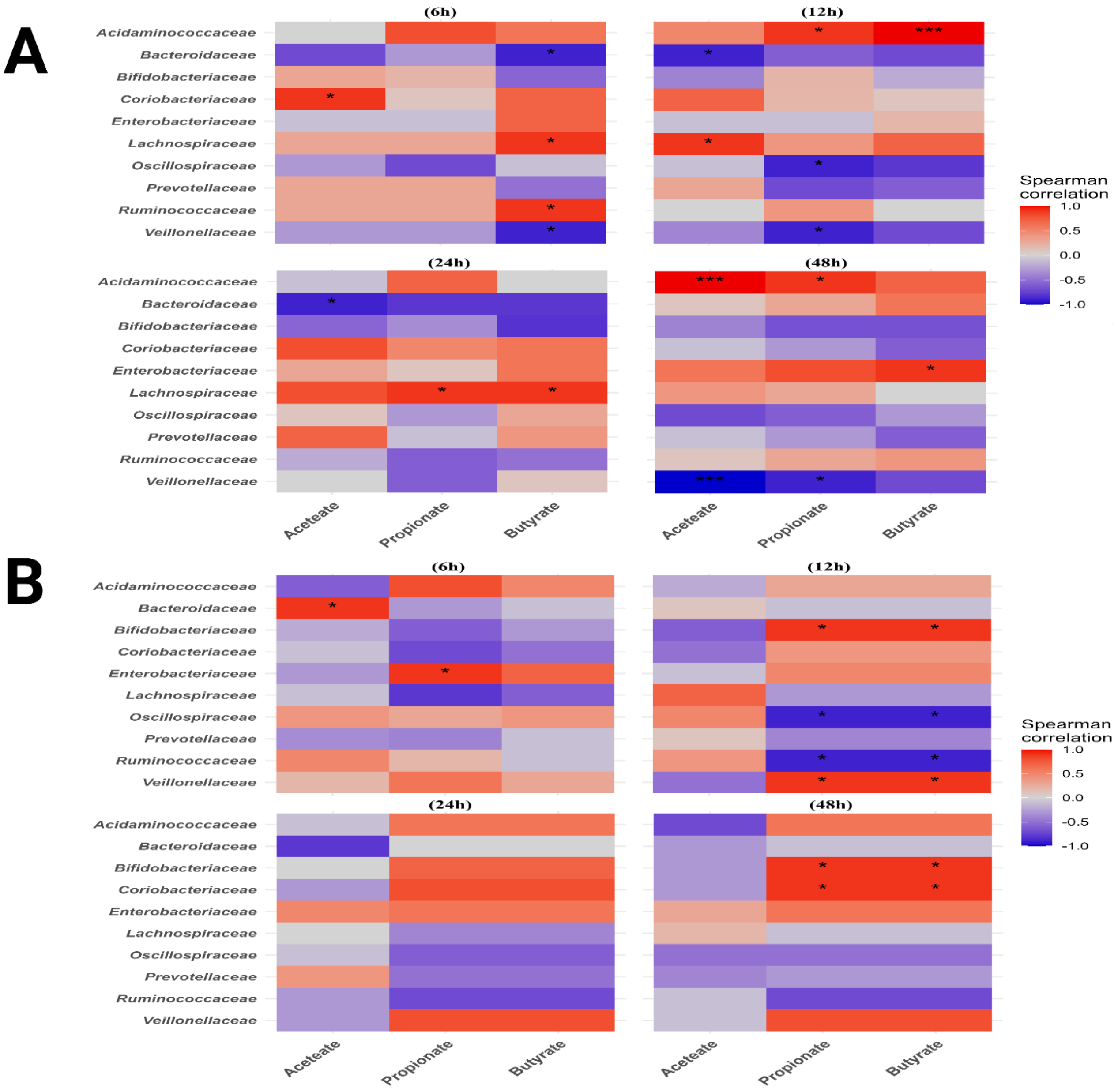

Fig. S1. Correlation heatmaps depict Spearman's correlation coefficients between the most abundant bacterial families and SCFAs in Control (A) and Female (B) simulators at four-time points (6h, 12h, 24h, and 48h). Dark red indicates strong positive correlations, while dark blue indicates strong negative correlations. Statistical significance is denoted as *$p < 0.05$, **$p \leq 0.01$, and ***$p \leq 0.001$.
